# Report of the Board of Health of Philadelphia, 1860

**Published:** 1861-06

**Authors:** 


Report of the Board of Health of Philadelphia for 1860.—Sanitary and Statistical. In accordance with an Act of the Legislature, approved March 8th, 1860, for the Registration of Births, Marriages and Deaths. Philadelphia: 1861.

Want of space, in the present number, alone prevents us making the receipt of this pamphlet, the occasion for some much-needed remarks on the barbarous condition of this most important subject in our State, *i.e.*, the registration-laws—the collection, under statute requirement and regulation, of those vital and mortuary statistics by which, alone, can the laws governing human existence be determined. So far as we are informed, marriages only are made the subject of registration in Illinois; if there is any law relative to the registration of deaths or even for the issue of certificates as to cause, they have alike fallen into disuse; while births would seem to be too common-place occurrences to merit any attention. But, given the enactment of judicious registration-laws of births, marriages and deaths, and their faithful enforcement, it would fall short of the requirements of the progress of the age; and we need, and must have, in addition, a *registration of sickness*—a branch which, to the shame of the profession, be it said, is and always has been entirely neglected—as though it concerned us to know the chances of the one death which must come to each, more than the probabilities of the multitude of diseases, which might be guarded against by a thorough, accurate knowledge of their habitats, times of greatest prevalence and the attendant circumstances which favor their eduction. The first without the last, is much as though the Surgeon-General should give us only the list of killed, omitting all mention of the wounded, in his casualty list.

The Report is a valuable one, and will, in the future, be of much use as a basis of statistics of the greatest importance.

